# Tidal Volume Challenge to Assess Volume Responsiveness with Dynamic Preload Indices During Non-Cardiac Surgery: A Prospective Study

**DOI:** 10.3390/jcm14010101

**Published:** 2024-12-27

**Authors:** Panagiota Griva, Emmanouil I. Kapetanakis, Orestis Milionis, Konstantina Panagouli, Maria Fountoulaki, Tatiana Sidiropoulou

**Affiliations:** 1 Second Department of Anesthesiology, Attikon University Hospital, National and Kapodistrian University of Athens, 12461 Athens, Greece; pgriva@med.uoa.gr (P.G.); orestismilionis@gmail.com (O.M.); kpana@med.uoa.gr (K.P.); mafountoulaki@gmail.com (M.F.); 2 Department of Thoracic Surgery, Attikon University Hospital, National and Kapodistrian University of Athens, 12461 Athens, Greece; emmanouil.kapetanakis@gmail.com

**Keywords:** tidal volume challenge, hemodynamic indices, pulse pressure variation, stroke volume variation, fluid responsiveness, responders, non-responders

## Abstract

**Background/Objectives**: The aim of this study is to assess whether changes in Pulse Pressure Variation (PPV) and Stroke Volume Variation (SVV) following a VtC can predict the response to fluid administration in patients undergoing surgery under general anesthesia with protective mechanical ventilation. **Methods**: A total of 40 patients undergoing general surgery or vascular surgery without clamping the aorta were enrolled. Protective mechanical ventilation was applied, and the radial artery was catheterized in all patients. The protocol began one hour after the induction of general anesthesia and the stabilization of hemodynamic parameters. The parameters PPV_6_ and SVV_6_ were recorded during ventilation with a Vt of 6 mL/kg Ideal Body Weight (IBW) (T1). Then, the Vt was increased to 8 mL/kg IBW for 3 min without changing other respiratory parameters. After the VtC, the parameters PPV_8_ and SVV_8_ (T2) were recorded. After the stabilization of hemodynamic parameters, volume expansion (VE) was administered with colloid fluid of 6 mL/kg IBW. Parameters before (T3) and 5 min after fluid challenge (T4) were recorded. The change in the Stroke Volume Index (SVI) before and after VE was used to indicate fluid responsiveness. Patients were classified as fluid responders (SVI ≥ 10%) or non-responders (SVI < 10%). **Results**: The parameter ΔPPV(6–8) demonstrated good predictive ability to predict fluid responsiveness, evidenced by an Area Under the Curve (AUC) of 0.86 [95% Confidence Interval (CI) 0.74 to 0.95, *p* < 0.0001]. The threshold of ΔPPV(6–8) exceeding 2% identified responders with a sensitivity of 83% (95% CI 0.45 to 1.0, *p* < 0.0001) and a specificity of 73% (95% CI 0.48 to 1.0, *p* < 0.0001). The parameter ΔSVV(6–8) also revealed good predictive ability, reflected by an AUC of 0.82 (95% CI 0.67 to 0.94, *p* < 0.0001). The criterion ΔSVV(6–8) greater than 2% pinpointed responders with a sensitivity of 83% (95% CI 0.71 to 1.0, *p* < 0.001) and a specificity of 77% (95% CI 0.44 to 1.0, *p* < 0.001). **Conclusions**: This study demonstrates that VtC possesses good predictive ability for fluid responsiveness in patients undergoing general surgery.

## 1. Introduction

The primary objective of perioperative fluid administration is to enhance cardiac output (CO) to improve end-organ perfusion (e.g., heart, brain, kidneys) [1]. This is achieved when a fluid bolus augments venous return, thereby optimizing the heart’s position on the Frank–Starling curve and increasing Stroke Volume (SV) [1]. The Frank–Starling curve demonstrates that, up to a certain point, an increase in end-diastolic volume leads to an increase in SV [2]. Assessing volume status and deciding if intravascular volume expansion will benefit end organs is complex. Inappropriate fluid administration can cause harm and increase mortality while avoiding fluids in hypovolemic patients is also detrimental. Excessive fluid administration is particularly harmful, as it can exacerbate conditions, such as cardiac failure and pulmonary edema [1]. Thus, fluid administration should be carefully evaluated for its potential benefits and risks [3]. Static parameters and dynamic parameters are the most common methods used to assess volume status [4]. Static parameters (i.e., Mean Arterial Pressure, Central Venous Pressure, Mixed Venous Oxygen Saturation, Pulmonary Capillary Wedge Pressure/Pulmonary Artery Occlusion Pressure) are indicators that provide information at a single point in time rather than reflecting dynamic changes. Specifically, a static indicator reflects preload at a particular point on the Frank–Starling curve but does not demonstrate the ability to traverse or reflect changes along the curve [5]. However, it has been demonstrated that static parameters fail to reliably predict fluid responsiveness [6]. The evaluation of dynamic parameters [i.e., Stroke Volume Variation (SVV), Pulse Pressure Variation (PPV), and the Plethysmographic Variability Index (PVI)] involves examining how physiological responses change due to cardiopulmonary interactions [7,8]. When intrathoracic pressure rises during inspiration, venous return to the right heart is temporarily reduced, decreasing right ventricular preload and, after a short delay, left ventricular output. In hypovolemic states, the ventricles operate on the steep portion of the Frank–Starling curve, where SV is highly sensitive to changes in preload, leading to more pronounced variations in PPV and SVV. Conversely, in euvolemic or hypervolemic states, the flatter portion of the curve results in smaller variations [8,9]. Dynamic indices are significantly more accurate compared with all other methods in predicting volume responsiveness [7,8,9]. Conventionally, the threshold for fluid responsiveness has been set at SVV, exceeding 13% [10], whereas PPV can anticipate fluid responsiveness using a cutoff of 13% [11]. Dynamic tests for fluid responsiveness apply the Frank–Starling law to evaluate whether a patient will benefit from increased fluid volume. This evaluation can be carried out using two primary methods: the fluid challenge and respirophasic variation. The fluid challenge approach may be either irreversible, involving the infusion of 250 to 500 mL of crystalloid solution over 10 min [12], or reversible such as the passive leg raise maneuver [13]. The respirophasic variation approach relies on physiological interactions with positive pressure ventilation and its effect on SV. Recently, adopting a lung-protective ventilation strategy involving a tidal volume (Vt) of less than 8 mL/kg has been linked to improved patient outcomes and is now recommended as a standard practice in the operating room [14]. However, this reduction in Vt can compromise the accuracy of dynamic indices like PPV and SVV. To address this limitation, it is necessary to use functional hemodynamic tests in order to increase the right ventricular preload [15]. Several researchers have suggested and validated the efficacy of the Tidal Volume Challenge (VtC) test in both surgical patients and critically ill patients in the Intensive Care Unit [16,17,18]. For mechanically ventilated patients, this test involves a temporary increase in Vt from 6 to 8 mL/kg IBW to observe changes in PPV [16]. Both ventilatory parameters (Vt, PEEP, chest and lung compliance) and cardiovascular variables (heart rate, ventricular function, and cardiac afterload) collectively impact SVV and PPV [19]. Nonetheless, it was found that Vt is a more significant determinant of pleural and pericardial pressures and right ventricular afterload than airway pressure [20].

The aim of this research is to investigate whether changes in PPV and SVV following a VtC can accurately predict fluid responsiveness in patients undergoing general or vascular surgery under general anesthesia and applying protective mechanical ventilation.

## 2. Materials and Methods

### 2.1. Participants and Eligibility Criteria

The study protocol was approved by the Institutional Ethics Committee of Attikon University Hospital of Athens, Greece, EU (557/4-11-2021), and was also prospectively registered at ClinicalTrials.gov registry (NCT: 05254951). After written informed consent was obtained from each patient, the study protocol was performed in patients who underwent moderate-to-high-risk non-cardiac surgery that required continuous invasive blood pressure monitoring intraoperatively. The inclusion criteria in the study were as follows: adults undergoing general or vascular surgery without clamping of the aorta, which required arterial cannulation and invasive blood pressure monitoring during surgery. The expected duration of the operation was equal to or greater than 90 min. Patients were excluded from the study if they manifested preoperative arrhythmia or newly emergent arrhythmia after anesthesia induction, reduced left (EF < 40%) or right systolic function, BMI > 30 kg/m^2^, the preoperative use of beta-blockers or chronic obstructive pulmonary disease with an FEV1 < 60% predicted volume.

### 2.2. Anesthetic Management

Patients were transferred to the operating room, where standard monitoring was applied. A radial arterial cannula was inserted for invasive monitoring of the arterial blood pressure prior to anesthesia induction. The catheter was connected to a transducer, which was connected both to the HemoSphere monitor (Edwards Lifesciences, Irvine, CA, USA) and to the operating room monitor (GE-Healthcare, Chicago, IL, USA). The arterial waveform was analyzed by the HemoSphere monitor in order to calculate other hemodynamic parameters such as Pulse Pressure Variation (PPV), Stroke Volume Variation (SVV), Stroke Volume Index (SVI), Cardiac Index (CI), MAP (Mean Arterial Pressure), and heart rate (HR).

The intraoperative anesthetic management was carried out by an independent clinician, who was informed of the study protocol but was not otherwise involved in the study. All patients received general anesthesia according to standard practice. Propofol (1.5–2.5 mg/kg), rocuronium (0.6 mg/kg), and opioids (fentanyl or remifentanil) were administrated for anesthesia induction. Maintenance of anesthesia was performed with sevoflurane at 1.5–2.5% in an oxygen/air mix and/or intravenous fentanyl bolus and/or continuous IV infusion of remifentanil at a rate of 0.02–0.2 mcg/kg/min. After tracheal intubation, mechanical positive pressure ventilation was applied with tidal volume = 6 mL/kg of Ideal Body Weight (IBW) and Positive End Expiratory Pressure (PEEP) = 5 cm H_2_O The respiratory rate and I:E ratio were adjusted accordingly to maintain End-Tidal Carbon Dioxide (EtCO_2_) levels between 35 and 40 mmHg. The IBW was calculated according to the formula x + 0.91 [height (in cm) − 152.4], where x = 50 for men and x = 45.5 for women [21].

### 2.3. Study Protocol

**Figure 1:**
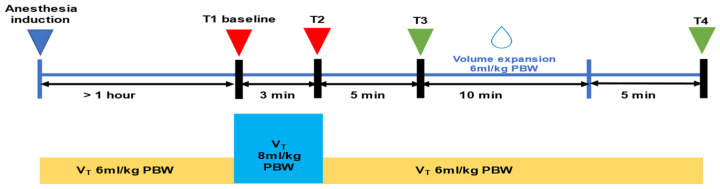
Schematic representation of the protocol. PBW = Predicted Body Weight; Vt = tidal volume.

A schematic representation of the protocol is shown in Figure 1. The study protocol took place one hour after the induction of general anesthesia and when the hemodynamic parameters were stable [changes in MAP < 10% for 5 min]. While the applied tidal volume was Vt = 6 mL/kg IBW (T1 baseline), the parameters MAP, SVI, CI, PIP (Peak Inspiratory Pressure), Cdyn (Dynamic Compliance), PPV6, and SVV6 were recorded. Then, the tidal volume increased to Vt = 8 mL/kg IBW (VtC) for 3 min without changing the other respiratory parameters. At the end of the VtC (T2), the parameters MAP, SVI, CI, PIP, Crs, PPV8, and SVV8 were recorded again, and the tidal volume decreased to 6 mL/kg. Changes in PPV and SVV values were calculated as follows: ΔPPV(6−8) = PPV8 − PPV6 and ΔSVV(6−8) = SVV8 − SVV6. The percentage changes in PPV and SVV [ΔPPV(6−8) (%) and ΔSVV(6−8) (%)] were also calculated. When the hemodynamic parameters were stable, at least 10 min after VtC, volume expansion (VE) was performed over 20 min. Colloid fluid (Gelofusine^®^) was administrated at a dose of 6 mL/kg IBW. The parameters MAP, SVI, CI, PIP, Cdyn, PPV8, and SVV8 were recorded again before (T3) and 5 min after the fluid challenge (T4). The difference in the SVI value before and after VE (ΔSVIVE) was an indicator of fluid response. Patients were divided into two groups (responders and non-responders) depending on the percentage of change in SVI. If ΔSVIVE ≥ 10%, the patients respond to volume expansion (responders). If ΔSVIVE < 10%, the patients did not respond to volume expansion (non-responders). The choice of a 10% variation in SVIVE as the threshold for identifying fluid responders is based on established clinical practice and the literature, where a 10% change has been widely validated as a reliable marker of fluid responsiveness [22,23,24,25].

### 2.4. Sample Size Calculation

The sample size was determined based on the hypothesis that ΔPPV(6–8) can predict response to fluid administration, assuming an Area Under the Curve (AUC) of 0.75, compared to the alternative null hypothesis (AUC = 0.5). To detect a difference of 0.25 between these AUC values, with a type I error rate of 0.05 and a desired study power of 0.80, it was calculated that a minimum of 38 patients would be required. To account for potential patient dropouts, this number was rounded to 40. The sample size calculation was performed using GPower 3.1.2 for Windows (Heinrich Heine University, Düsseldorf, Germany).

### 2.5. Statistical Analysis

The normality of the variables was assessed using the Shapiro–Wilk test. Data are presented as the mean ± standard deviation, median (interquartile range [IQR]), or absolute value (percentage). For continuous variables, the Mann–Whitney U test was employed, while the χ2 test was used for categorical variables. Correlations between the variables were analyzed using Spearman’s correlation test.

To evaluate the predictive capacity of dynamic indicators for fluid responsiveness, receiver-operating characteristic (ROC) curves were utilized. The area under the ROC curve (AUC) was calculated and compared using the DeLong method. Briefly, the interpretation of the AUC was as follows: AUC = 0.5 indicates an unreliable test; AUC = 0.6–0.69 suggests poor predictive ability; AUC = 0.7–0.79 indicates moderate predictive ability; AUC = 0.8–0.89 reflects good predictive ability; AUC = 0.9–0.99 indicates excellent predictive ability; and AUC = 1.0 represents a test with perfect prediction. For each variable, an optimal threshold value was identified to maximize the Youden Index (sensitivity + specificity − 1). A gray zone for dynamic preload indices was established to account for possible overlap between responders and non-responders, defined by a lower cut-off value capturing 90% of negative fluid challenge responses and a higher cut-off value predicting positive fluid challenge responses in 90% of cases.

## 3. Results

### 3.1. Study Population

From March 2022 to September 2023, 40 surgical patients were considered eligible for enrollment in the study. The study protocol was successfully applied to each patient without complications. Patients were stratified into two distinct cohorts (responders and non-responders) based on the percentage variation in SVI. If ΔSVIVE is greater than or equal to 10%, the patients exhibit a positive response to volume expansion (responders). Conversely, if ΔSVIVE is less than 10%, the patients demonstrate an absence of response to volume expansion (non-responders). Among the 40 patients included in the final analysis, 19 patients (47.5%) were responders, and 21 (52.5%) were non-responders.

The Mann–Whitney U test was applied to explore potential statistically significant variations in the sample. There were no statistically significant (*p* < 0.05) differences between the responders and non-responders, indicating that both their demographic characteristics and procedural characteristics are comparable. Demographic data and intraoperative characteristics are presented in Table 1.

### 3.2. Effects of VtC and VE on Hemodynamic and Respiratory Variables

The evolution of hemodynamic and respiratory variables before and after the VtC, and before and after VE, in both responders and non-responders, is shown in Table 2, Table 3, Table S1 and Table S2, respectively.

At the time point T1, there was no statistically significant difference in the parameters PPV (*p* = 0.859) and SVV (*p* = 0.805), nor in MAP (*p* = 0.838), between responders and non-responders (Table 2). However, following the application of VtC, a statistically significant difference emerged between responders and non-responders with respect to the aforementioned hemodynamic parameters, specifically PPV (*p* = 0.005), SVV (*p* = 0.006), and MAP (*p* = 0.048) (Table 2).

The VtC application resulted in a significant augmentation in both PPV and SVV among the responders, with percentages escalating from 8.0% [6.3 to 10.0] to 11.0% [10.0 to 12.8] (*p* < 0.00045) and from 8.0% [6.3 to 9.0] to 10.0% [9.0 to 12.0] (*p* < 0.00045) for PPV and SVV, respectively (Table 2). In contrast, no statistically significant alteration was detected among non-responders, as the percentages of PPV and SVV remained stable, with values ranging from 7.5% [5.3 to 10.8] to 7.0% [5.0 to 9.8] (*p* = 0.732), and from 7.0% [5.3 to 9.8] to 7.0% [5.0 to 9.0] (*p* = 0.934) for PPV and SVV, respectively (Table 2).

Following VE, a significant reduction in PPV and SVV was observed exclusively in responders. Specifically, PPV decreased from 9.0% (8.0 to 13.3) to 7.5% (5.0 to 9.0) (*p* < 0.05), and SVV decreased from 8.5% (7.3 to 10.8) to 7.5% (5.3 to 8.0) (*p* < 0.027). As anticipated, SVI showed a statistically significant increase after VE only in responders, increasing from 39.0 mL/min^2^ (32.5 to 44.8) to 46.5 mL/min^2^ (39.3 to 51.8) (*p* < 0.031).

Regarding the ventilatory parameters, no statistically significant differences were observed between the responders and non-responders across the designated time intervals (Tables S1 and S2).

### 3.3. Prediction of Fluid Responsiveness

**Figure 2:**
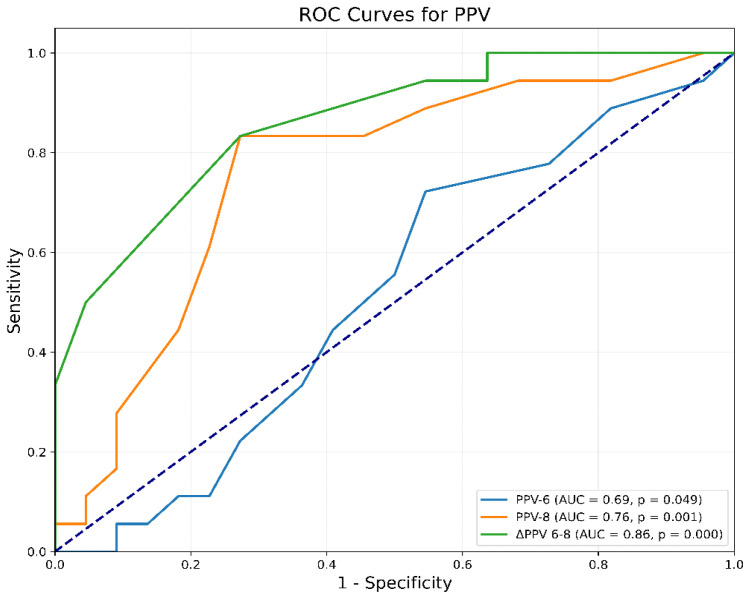
Receiver-operating characteristic curve comparing the ability of PPV to predict fluid responsiveness: ΔPPV(6–8) = change in PPV after increasing Vt from 6 to 8 mL/kg IBW; PPV6 = PPV at Vt 6 mL/kg IBW; PPV8 = PPV at Vt 8 mL/kg IBW; PPV = Pulse Pressure Variation; Vt = tidal volume.

According to the analysis of the receiver-operating characteristic (ROC) curve, the parameter ΔPPV(6–8) demonstrated a good predictive ability to predict fluid responsiveness, evidenced by an Area Under the Curve (AUC) of 0.86 [95% Confidence Interval (CI) 0.74 to 0.95, *p* < 0.0001]. The threshold of ΔPPV(6–8) exceeding 2% identified responders with a sensitivity of 83% (95% CI 0.45 to 1.0, *p* < 0.0001) and a specificity of 73% (95% CI 0.48 to 1.0, *p* < 0.0001). The Youden Index was 0.56. Additionally, PPV6 and PPV8 were found to possess limited efficacy in predicting fluid responsiveness, as demonstrated by an AUC of 0.69 (95% CI, 0.33 to 0.69, *p* = 0.05) and 0.76 (95% CI 0.59 to 0.89, *p* < 0.0006), respectively (Figure 2).

**Figure 3:**
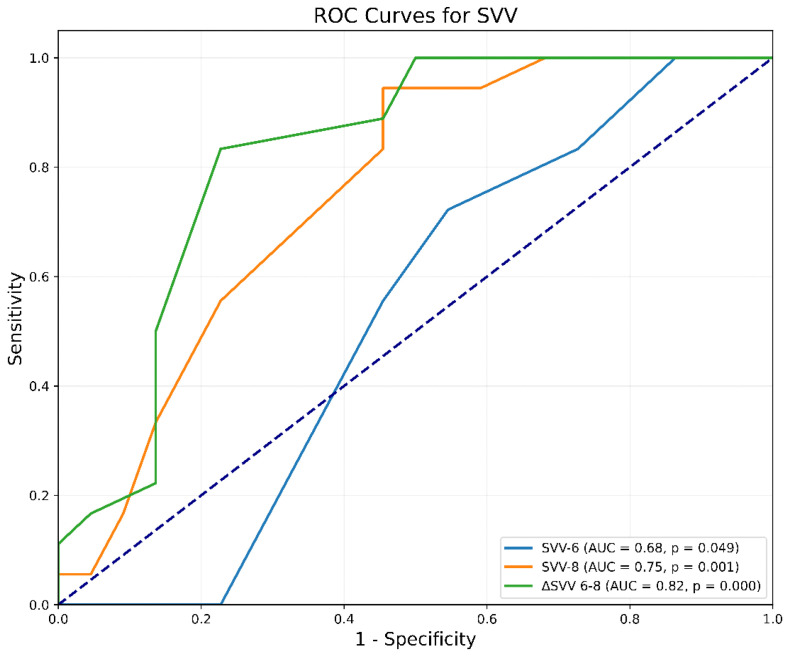
Receiver-operating characteristic curves comparing the ability of SVV to predict fluid responsiveness. ΔSVV(6–8) = change in SVV after increasing Vt from 6 to 8 mL/kg IBW; SVV6 = SVV at Vt 6 mL/kg IBW; SVV8 = SVV at Vt 8 mL/kg IBW; SVV = Stroke Volume Variation; Vt = tidal volume.

Additionally, ΔSVV(6–8) also revealed good predictive ability, reflected by an AUC of 0.82 (95% CI 0.67 to 0.94, *p* < 0.0001). The criterion of ΔSVV(6–8) greater than 2% pinpointed responders with a sensitivity of 83% (95% CI 0.71 to 1.0, *p* < 0.001) and a specificity of 77% (95% CI 0.44 to 1.0, *p* < 0.001). The Youden Index was 0.61. The predictive abilities of SVV6 and SVV8 were considerably lower as the AUC values were 0.68 (95% CI 0.33 to 0.69, *p* = 0.049) and 0.75 (95% CI 0.59 to 0.89, *p* < 0.0001), respectively (Figure 3).

The AUC metrics for ΔPPV(6–8) and ΔSVV(6–8) exhibited statistically significant elevations when compared to those of PPV and SVV at tidal volumes of 6 mL/kg IBW and 8 mL/kg IBW, respectively (Table 3).

Following the VtC, the subsequent changes in the hemodynamic parameters, Cardiac Index (CI) and Stroke Volume Index (SVI), were evaluated. The interpretation of the resulting curves indicates that the tests demonstrate low predictive accuracy, with AUC values of 0.47 (95% CI 0.27 to 0.66, *p* = 0.75) and 0.49 (95% CI 0.32 to 0.68, *p* = 0.94), for ΔCI6-8 (Figure S1), and ΔSVI6-8 (Figure S2), respectively.

### 3.4. Gray-Zone Approach for ΔPPV(6–8) and ΔSVV(6–8)

The gray-zone approach was used to illustrate the inconclusive region of hemodynamic changes for predicting fluid responsiveness. For ΔPPV(6–8), the gray zone ranged between 1.6% and 2.4%, encompassing 11 patients, including 6 responders and 5 non-responders (Table 4). Similarly, the gray zone for ΔSVV(6–8) ranged from 1.6% to 3.4%, involving 13 patients, with 11 responders and 2 non-responders.

## 4. Discussion

This study demonstrated that VtC possesses good predictive ability for fluid responsiveness, as evidenced by the absolute changes in the PPV and SVV parameters observed during VtC (ΔPPV(6–8) and ΔSVV(6–8)). Furthermore, our analysis identified that a threshold exceeding 2% for both aforementioned parameters accurately distinguishes between patients likely to respond to fluid administration and those unlikely to exhibit fluid responsiveness. Additionally, our research established that the predictive value of PPV and SVV is reduced when patients are ventilated with protective mechanical ventilation using a Vt of 6 mL/kg IBW, compared to larger tidal volumes of 8 mL/kg IBW.

The Vt increment, as facilitated by the VtC, has the capacity to modify intrapleural pressure, consequently influencing venous return and the filling pressures of the right atrium. This biomechanical effect may transiently diminish the Stroke Volume [26]. PPV and SVV are dynamic parameters utilized to evaluate the fluctuations in SV and pulse pressure throughout the respiratory cycle, specifically affected by alterations in intrathoracic pressures induced by mechanical ventilation. These parameters exhibit a closer association with preload dependency and demonstrate a heightened sensitivity to the acute variations in venous return that occur with increased tidal volume [27]. Specifically, increasing the average pleural pressure with a rise in Vt is expected to reduce venous return, thereby shifting the Frank–Starling curve to the left [20]. This reduction in venous return decreases right ventricular preload, increases right ventricular afterload, and decreases left ventricular preload, which, in turn, reduces left ventricular work and increases PPV [20].

Our study demonstrated that performing a VtC significantly enhances the reliability of the PPV and SVV tests, as evidenced by our findings. This finding aligns with the results of previous studies by Botros et al. [24] and Tang et al. [25]. In Tang et al.’s study [25], the AUC of ΔPPV(6–8) after VtC was 0.85 [*p* < 0.0001], while Botros et al. [24] reported an AUC of ΔPVI6–8 of 0.86 [*p* < 0.001]. However, the AUCs of ΔPPV(6–8) found in these studies were lower than those reported in other studies, such as those by Myatra et al. [16] and Jun et al. [18]. Their studies demonstrated AUCs of ΔPPV(6–8) of 0.99 [*p* < 0.0001], respectively. The variation in AUCs across these studies may be explained by differences in the definition of fluid responsiveness. For example, a 10% increase in SVI following VE was employed as a criterion for distinguishing between responders and non-responders in this study, as well as in the studies conducted by Botros et al. [24] and Tang et al. [25] In Myatra’s study [16], an increase in CI by more than 15% was the threshold for discriminating responders from non-responders, whereas in Jun’s study [18], an increase in the CI of 15% or more was the defined limit.

The respirophasic variation requires normal heart rhythm and a tidal volume of 8 mL/kg IBW or more [12]. A reduced Vt can compromise the reliability of dynamic indices such as PPV and SVV [28]. Lower tidal volumes can result in the diminished amplitude of the physiological fluctuations that these indices rely on, potentially leading to less accurate measurements and, consequently, less reliable predictions regarding fluid responsiveness [29]. This research yielded analogous results. PPV6 was found to possess limited efficacy in predicting fluid responsiveness in comparison with PPV8. The predictive abilities of SVV6 and SVV8 were considerably lower.

The VtC failed to enhance the predictive efficacy of CI and SVI. The Cardiac Index is influenced by several factors, including heart rate and Stroke Volume. Stroke Volume is affected by three variables—contractility, afterload, and preload [30]. The VtC can momentarily reduce the Stroke Volume, distorting CI measurements during or immediately after the challenge [31]. In essence, CI reflects more global, continuous trends rather than the subtle, transient changes in Stroke Volume caused by the VtC, making it less responsive in the short term. The SVI measurement, being more static, may not immediately reflect the fluid responsiveness or preload changes because it captures a broader hemodynamic response rather than moment-to-moment variations [32]. Thus, SVI is a more average and less responsive parameter to rapid shifts in preload. SVI reflects overall cardiac performance but lacks real-time responsiveness to the small, transient changes in preload caused by a VtC. On the other hand, SVV and PPV are dynamic indices that provide better real-time assessments of how Stroke Volume varies with respiratory cycles, making them more reliable after a VtC [33].

The establishment of an optimal threshold for predicting fluid responsiveness subsequent to a VtC is crucial to affirm its clinical applicability. The current literature encompasses a broad range of threshold values and methodological frameworks, which may impact the sensitivity and specificity related to VtC. In this research, we demonstrated that a 2% increase in PPV after VtC is optimal for accurately distinguishing between responders and non-responders. These results are consistent with the findings of Xu et al. [34], as a threshold of 2% was also proposed. Research by Myatra et al. [16], Shi et al. [35], and Elsayed et al. [36] has determined a higher optimal cutoff value for PPV at 3.5%. In contrast, further studies by Tang et al. [25] and Jun et al. [18] have identified even lower observed thresholds of 1%. A lower cutoff value for PPV in the context of VtC generally signifies that a smaller increase in PPV is sufficient to predict fluid responsiveness before it becomes more pronounced. This can facilitate earlier intervention and more precise fluid management [37]. However, while a lower cutoff enhances sensitivity, it may also increase the likelihood of false positives, potentially leading to unnecessary fluid administration in patients who may not actually benefit from it [38].

Regarding ΔSVV(6-8), our research identified an optimal threshold of 2%, which aligns with the findings of the study by Jun et al. [18]. Additionally, Myatra’s research [16] indicated a higher optimal threshold of 2.5%. This discrepancy may be attributed to both the smaller patient sample and differences in study design. Smaller sample sizes can yield fewer stable estimates of the optimal threshold. Specifically, Myatra’s study [16] included 22 patients, whereas our study and Jun’s study [18] recruited 40 and 38 patients, respectively. Additionally, variations in the type of VtC employed may influence the identified thresholds. For instance, Myatra’s study [16] augmented the VtC for 1 min, while in our study and Jun’s study [18], the VtC was extended for 3 min.

Following the VtC, there was a significant increase in PPV and SVV in responders, with PPV rising from 8.0% to 11.0% (*p* < 0.00045) and SVV from 7.0% to 10.0% (*p* < 0.00045). In contrast, non-responders showed no significant changes in PPV or SVV, with values remaining stable (*p* = 0.732 for PPV and *p* = 0.934 for SVV). This finding is consistent with the existing literature. For instance, the study by Messina et al. [39] demonstrated that the VtC application significantly increased both PPV and SVV in responders (from 6.3% [4.1 to 7.5] to 10.3% [7.6 to 12.7], *p* < 0.0001, and from 7.3% [5.3 to 9.4] to 10.8% [7.8 to 12.8], *p* < 0.0001, for PPV and SVV, respectively), whereas no such increase was observed in non-responders. A notable elevation in PPV and SVV was observed solely among responders when VtC occurred due to the fact that these responders remained in a condition in which their cardiac function exhibited heightened sensitivity to fluctuations in the preload. Patients who exhibit responsiveness to volume therapy are situated on the “steep” segment of the Frank–Starling curve [40]. This indicates that their SV and CO display a marked sensitivity to variations in the preload. In patients undergoing mechanical ventilation, the fluctuations in intrathoracic pressure throughout the respiratory cycle significantly affect venous return and, as a result, impact SV [41]. In individuals classified as fluid responders, these fluctuations are markedly accentuated due to their physiological state, wherein Stroke Volume demonstrates heightened sensitivity to variations in venous return (i.e., they are preload-sensitive). In non-responders, the heart operates on the flat portion of the Frank–Starling curve, meaning that additional fluid does not significantly increase SV or CO [42]. These patients are less sensitive to changes in the preload, and as a result, respiratory-induced variations in SVV and PPV are minimal or unchanged after VtC.

The increase in MAP observed after the VtC exclusively in responders highlights a hemodynamic improvement associated with fluid responsiveness. This occurs because responders, who are likely operating on the steep portion of the Frank–Starling curve, experience an increase in SV following the VtC due to an improved preload [42]. An enhanced SV leads to an increased CO, which, in turn, raises MAP. In non-responders, the absence of a significant MAP change suggests that their preload is already optimized or that other factors, such as reduced myocardial contractility or vascular compliance, limit their hemodynamic response. This reinforces the role of VtC not only as a diagnostic tool to identify fluid responsiveness but also as a mechanism to uncover differences in the cardiovascular reserve between patient subgroups.

In cases where VtC fails to provoke notable alterations owing to variables such as diminished lung compliance, this accentuates the necessity of comprehending the physiological constraints of this methodology in specified patient demographics. Diminished lung compliance (i.e., Acute Respiratory Distress Syndrome) may attenuate the transference of airway pressure fluctuations to the cardiac system, consequently restricting the capacity of VtC to discern fluid responsiveness. In such scenarios, it becomes imperative to contemplate adjunctive methodologies, such as recruitment maneuvers or tailored PEEP titration, which may enhance pulmonary mechanics and augment the efficacy of VtC [43]. Moreover, the incorporation of advanced hemodynamic monitoring devices can yield more detailed information to evaluate preload responsiveness in patients exhibiting complex respiratory mechanics. This underscores the imperative for the meticulous interpretation of VtC outcomes within the context of individual patient characteristics and their overarching hemodynamic and respiratory profile.

This study has several limitations. Initially, the study population consisted of a specific group of carefully selected patients who underwent general surgery or vascular surgery without clamping of the aorta. Obese patients (BMI > 30 kg/m^2^), patients with decreased systolic function, and chronic obstructive disease with FEV1 < 60% of predicted volume were excluded from the study. Therefore, further studies should investigate whether a VtC is beneficial in such high-risk cases. Second, the sample size was from a single center. Multicenter studies should be performed to confirm these results. Third, in this study, fluid responsiveness was defined by a threshold of an increase in SVI greater than 10%. Utilizing a >10% threshold likely encompasses a broader spectrum of patients as responders, including those exhibiting mild-to-moderate increases in SVI. Fourth, the precision of the HemoSphere monitor is significantly contingent upon the caliber of the signal acquired from the arterial catheter. Artifacts associated with underdamping or overdamping, as well as those pertaining to the transmission of the signal, may adversely affect the dependability of the device. Fifth, colloid fluids were administrated to assess volume responsiveness. It is worth noting that when administered in equal quantities, colloid solutions exhibited approximately four times greater plasma expansion effects compared to crystalloid solutions [44]. Finally, in the present study, the tidal volume increased to 8 mL/kg IBW in 3 min. It is recommended that future research explores varying time intervals for tidal volume adjustments to determine whether this parameter influences the accuracy of the test.

## 5. Conclusions

In this single-center prospective study, we demonstrated that changes in PPV and SVV following a VtC exhibit good predictive accuracy for fluid responsiveness patients undergoing surgery under general anesthesia with protective mechanical ventilation. However, further research is required to evaluate the reliability of VtC across different types of surgeries and diverse surgical populations.

## Figures and Tables

**Table 1:** Demographic data and intraoperative characteristics.

	Responders (*n* = 19)	Non-Responders (*n* = 21)
Age (years)	64.9 ± 12.3	63.8 ± 12.7
Sex (M/F)	9/10	12/9
BMI (kg/m^2^)	25.2 [6.3]	25.9 [5.5]
IBW (kg)	62.6 ± 10.3	61.8 ± 7.9
ASA score	2.0 [0.8]	2.0 [1.0]
Preop Hb (g/dL)	11.5 ± 2.3	12.5 ± 1.6
Preop Cr (mg/dL)	0.81 ± 0.22	0.8 [0.4]
Baseline eGFR (mL/min/1.73 m^2^)	42.0 ± 8.9	47.5 ± 11.1
Comorbidities		
Hypertension	47%	53%
Coronary Artery Disease	5%	5%
COPD/Asthma	0%	11%
Cerebrovascular Disease	0%	5%
Diabetes Mellitus	26%	16%
Chronic Renal Disease	0%	5%
Malignancy	79%	90%
Perioperative data		
Surgery duration (minutes)	253.0 ± 115.7	200.0 [88.2]
Anesthesia duration (minutes)	284.0 ± 115.4	233.5 [108]
Epidural (Yes/No)	53%/47%	52%/48%
Total Crystalloids (mL)	3294.0 ± 1442.0	3000.0 [1850]
Fluids infused before VE (mL)	1200.0 [650]	1200.0 [375]
Sevoflurane (% min)	539.0 ± 237.0	490.0 [191.5]
Total Phenylephrine (mg)	8.3 [6]	4.8 [25.7]
Total Ephedrine (mg)	0.0 [9.4]	2.5 [17.5]
Intraoperative Diuresis (mL)	831.0 ± 475.0	600.0 [550]
Blood loss (mL)	525 [312.5]	400 [825]
In-hospital stay (days)	8.5 [6.8]	9.0 [14.0]
NSQIP score for any complication (%)	22.5 ± 0.09	17.4 ± 0.09
NSQIP score for serious complication (%)	19.2 ± 0.08	15.5 ± 0.08

Data represent mean ± SD; median [IQR] or numbers. For all parameters *p* > 0.05. Abbreviations: BMI: Body Mass Index; IBW: Ideal Body Weight; Hb: Hemoglobin; Cr: Creatinine; COPD: chronic obstructive pulmonary disease; eGFR: estimated Glomerular Filtration Rate; ASA: American Society of Anesthesiologists classification; NSQIP: National Surgical Quality Improvement Program; VE = volume expansion.

**Table 2:** Comparison of hemodynamic parameters between responders and non-responders at each time interval.

		Before VtC (T1)	After VtC (T2)	Before VE (T3)	After VE (T4)
HR (beats/min)	Responders	76 (68–85)	73 (59–80)	77 (67–82)	71 (66–79)
	Non-R	76 (62–78)	73.5 (66–81)	70 (65–81)	69.5 (63 to 78)
MAP (mmHg)	Responders	78 (71–87)	73 (59–79) *	83 (74–90) *	85 (78–93)
	Non-R	80 (69–87)	80 (69–91)	90 (83–99)	83 (74–96)
SVI (mL/min^2^)	Responders	38 (35–43.8)	36.5 (34–43)	39 (32.5–44.8)	46.5 (39.3–51.8) ^$^
	Non-R	43.5 (36.8–50.8)	47 (35.5–58)	50.5 (34.5–60)	42 (34.3–59.8)
CI (L/min/m^2^)	Responders	2.9 (2.6–3.5)	2.8 (2.5–3.4)	3.0 (2.2–3.6)	3.3 (2.7–4.1)
	Non-R	2.9 (2.5–3.5)	3.3 (2.5–3.7)	3.0 (2.5–3.7)	2.9 (2.2–3.8)
PPV (%)	Responders	8 (6.3–10) **	11 (10–12.8) **^,$$^	9 (8–13.3) *	7.5 (5–9) ^$^
	Non-R	7.5 (5.3–10.8)	7 (5–9.8)	6.5 (5–10.5)	6.5 (4–8.8)
SVV (%)	Responders	8 (6.3 to 9) **	10 (9–12) **^,$$^	8.5 (7.3–10.8) *	7.5 (5.3–8) ^$^
	Non-R	7 (5.3–9.8)	7 (5–9)	6 (5.3–9)	6 (5–9.5)
CVP (mmHg)	Responders	10 (9–12)	10 (7.3–11)	9.5 (7–11.8)	11 (9.3–12.8)
	Non-R	10 (8–13)	10 (8.3–11)	10.5 (6.3–12)	10 (8–13)

* *p* is significant at the <0.05 level or ** *p* < 0.01 between responders and non-responders (between groups). ^$^ *p* is significant at the <0.05 level or ^$$^ *p* < 0.01 before and after the VtC/VE challenge (intragroup). Values represent the median (IQR). Abbreviations: VtC: Tidal Volume Challenge; VE: volume expansion; HR: heart rate; MAP: Mean Arterial Pressure; SVI: Stroke Volume Index; CI: Cardiac Index; PPV: Pulse Pressure Variation; SVV: Stroke Volume Variation; CVP: Central Venous Pressure.

**Table 3:** Prediction of fluid responsiveness based on ROC curves.

	AUC (95% CI)	*p* Value	Youden Index	Cut-Off Value (%)	Sensitivity (%)	*p* Value	Specificity (%)	*p* Value
PPV6	0.69(0.33–0.69)	0.05	0.18	7	72	0.002	45	0.56
PPV8	0.76 (0.59–0.89)	0.0006	0.56	10	83	<0.0001	73	<0.001
ΔPPV(6–8)	0.86 (0.74–0.95)	<0.0001	0.56	2	83	<0.0001	73	0.0012
SVV6	0.68 (0.33–0.69)	0.049	0.18	7	72	<0.0001	45	0.56
SVV8	0.75 (0.59–0.89)	<0.0001	0.49	8	94	<0.0001	54	0.56
ΔSVV(6–8)	0.82 (0.67–0.94)	<0.0001	0.61	2	83	<0.0001	77	<0.0001

Abbreviations: PPV6 = pulse pressure variation during tidal volume at 6 mL/kg Ideal Body Weight (IBW); SVV6 = stroke volume variation during tidal volume at 6 mL/kg IBW; PPV8 = Pulse Pressure Variation during tidal volume at 8 mL/kg PBW; SVV8 = Stroke Volume Variation during tidal volume at 8 mL/kg PBW; ΔPPV(6–8) = change in value of PPV after tidal volume challenge; ΔSVV(6–8) = change in value of SVV after tidal volume challenge.

**Table 4:** Gray-zone for ΔPPV(6–8) and ΔSVV(6–8).

	Gray Zone (%)	Number of Patients in the Gray Zone (%)	Responders in the Gray Zone	Non-Responders in the Gray Zone	Gray-Zone Sensitivity (90% CI)	Gray-Zone Specificity (90% CI)
PPV6	4 to 16	36 (90%)	17/19	19/21	72	45
SVV6	5 to 14	37 (92.5%)	18/19	19/21	73	46
PPV8	6 to 13	26 (65%)	13/19	13/21	83	73
SVV8	8 to 13	25 (63%)	16/19	9/21	94	54
ΔPPV(6–8)	1.6 to 2.4	11 (27.5%)	6/19	5/21	83	73
ΔSVV(6–8)	1.6 to 3.4	13 (22.5%)	11/19	2/21	83	77

Abbreviations: VtC = Tidal Volume Challenge; CI Confidence Interval; PPV6 = Pulse pressure variation during tidal volume at 6 mL/kg Ideal Body Weight (IBW); SVV6 = Stroke Volume variation during tidal volume at 6 mL/kg IBW; PPV8 = Pulse Pressure Variation during tidal volume at 8 mL/kg PBW; SVV8 = Stroke Volume Variation during tidal volume at 8 mL/kg PBW; ΔPPV(6–8) = change in value of PPV after tidal volume challenge; ΔSVV(6–8) = change in value of SVV after tidal volume challenge.

## Data Availability

Data are contained within the article and Supplementary Materials.

## Supplementary Materials

The following supporting information can be downloaded at https://www.mdpi.com/article/10.3390/jcm14010101/s1. Figure S1: Receiver-operating characteristic curve comparing the ability of CI to predict fluid responsiveness; Figure S2: Receiver-operating characteristic curve comparing the ability of SVI to predict fluid responsiveness; Table S1: Comparison of ventilatory parameters between responders and non-responders at each time interval; Table S2: Comparison of ventilatory parameters after the application of VtC and VE in responders and non-responders.
