# Supplementary material for: Tidal Volume Challenge to Assess Volume Responsiveness with Dynamic Preload Indices During Non-Cardiac Surgery: A Prospective Study
Source: J Clin Med. 2024 Dec 27;14(1):101. doi: 10.3390/jcm14010101 (PMC11721188; doi:10.3390/jcm14010101)
Supplement: Supplementary file 1 [file jcm-14-00101-s001.zip › jcm-3308944-supplementary.pdf]

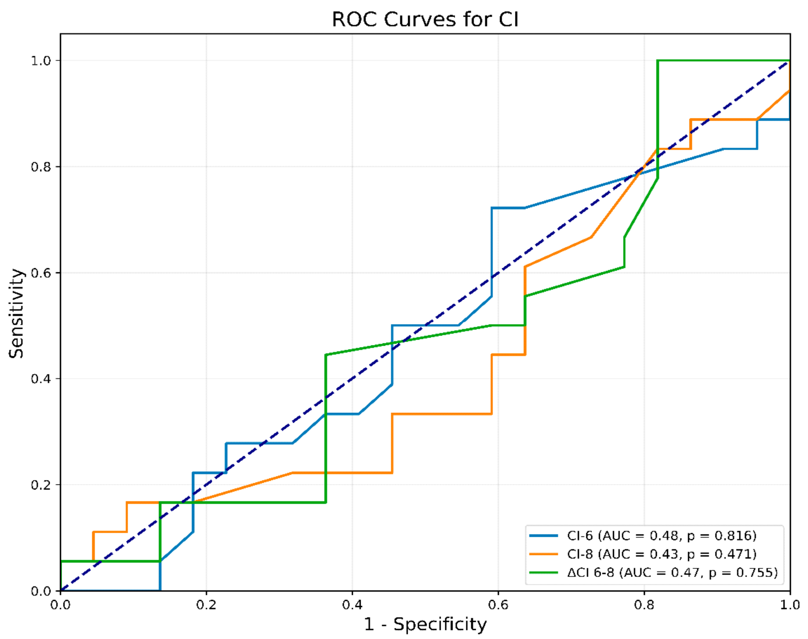

Figure S1: Receiver-operating characteristic curve comparing the ability of CI to predict fluid responsiveness. ΔCI_{6-8} = change in CI after increasing Vt from 6 to 8 mL/kg IBW, CI_6 = CI at Vt 6 mL/kg IBW,CI_8 = CI at Vt 8 mL/kg IBW, CI= Cardiac Index, Vt = tidal volume

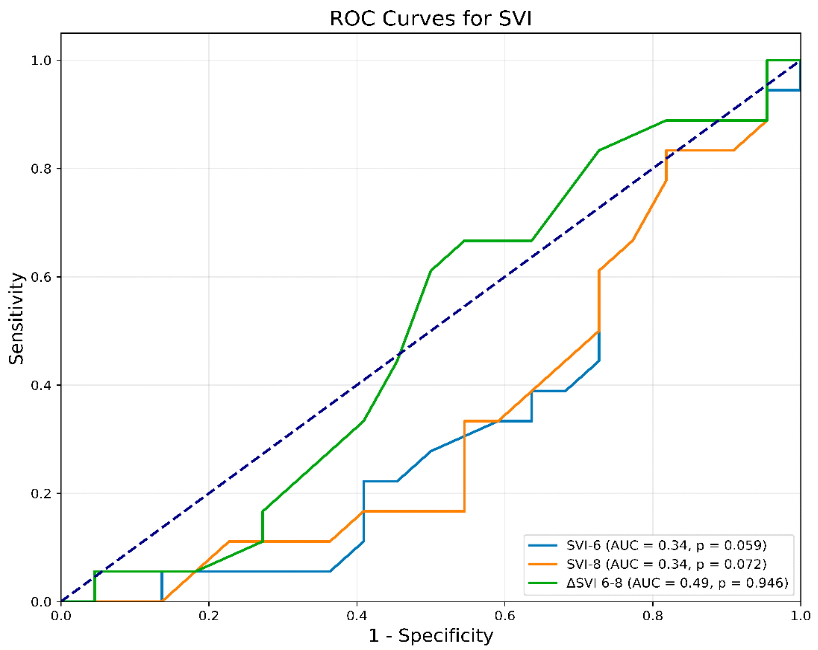

Figure S2: Receiver-operating characteristic curve comparing the ability of SVI to predict fluid responsiveness. $\Delta SVI_{6\text{–}8}$ = change in SVI after increasing Vt from 6 to 8 mL/kg IBW, SVI_6 = CI at Vt 6 mL/kg IBW, SVI_8 = SVI at Vt 8 mL/kg IBW, SVI= Stroke Volume Index, Vt = tidal volume

Table S1: Comparison of Ventilatory parameters between Responders and Non-Responders at Each Time Interval

	before VtC (T1)	P value	after VtC (T2)	P value	before VE (T3)	P value	after VE (T4)	P value
PIP (cmH_2O)								
Responders	17.5 (16.3 to 19.8)	0.115	20.5 (18.3 to 23.0)	0.420	18.0 (16.0 to 20.8)	0.193	18.0 (16.3 to 20.0)	0.325
Non-responders	16.5 (15.0 to 18.8)		20.0 (17.3 to 21.8)		17.0 (16.0 to 18.0)		18.0 (15.3 to 19.0)	
C_{dyn} (mL/cmH_2O)								
Responders	51.5 (34.8 to 62.2)	0.703	58.0 (42.5 to 66.0)	0.860	53.5 (37.8 to 65.9)	0.967	53.1 (46.1 to 60.8)	0.314
Non-responders	51.0 (46.0 to 56.8)		55.0 (49.0 to 63.3)		52.0 (42.5 to 58.0)		47.0 (39.3 to 56.8)	
Pplat (cmH_2O)								
Responders	13.5 (12.3 to 15.8)	0.210	15.0 (14.0 to 16.0)	0.358	14.0 (12.3 to 16.8)	0.209	14.5 (13.0 to 15.8)	0.231

Non-responders	13.0		14.5		13.0		13.5	
	(12.0 to 14.8)		(13.3 to 16.8)		(12.3 to 15.0)		(12.3 to 15.0)	
DP (cmH$_2$O)								
Responders	8.5		10.0		9.0		9.5	
	(7.3 to 10.8)	0.210	(9.0 to 11.0)	0.358	(7.3 to 11.8)	0.209	(8.0 to 10.8)	0.231
Non-responders	8.0		9.5		8.0		8.5	
	(7.0 to 9.8)		(8.3 to 11.8)		(7.3 to 10.0)		(7.3 to 10.0)	
Vt (mL)								
Responders	382.5		513.0		382.5		382.5	
	(325.0 to 410.0	0.881	(427.5 to 540.0)	0.892	(325.0 to 410.0)	0.902	(325.0 to 410.0)	
Non-responders	375.0		493.0		375.0		375.0	0.902
	(340.0 to 411.5)		(451.3 to 547.5)		(340.0 to 411.5)		(340.0 to 411.5)	
RR (breaths/min)								
Responders	20.0		20.0		20.0		19.5	
	(16.5 to 20.0)	0.385	(16.5 to 20.0)	0.321	(16.5 to 20.0)	0.685	(16.5 to 20.0)	0.814
Non responders	18.5		18.0		19.0		18.0	
	(16.3 to 20.0)		(16.3 to 20.0)		(18.0 to 20.0)		(16.5 to 20.0)	

$PaCO_2$ (mmHg)								
Responders	45.0		41.5		44.5		45.0	
	(44.0 to 47.0)	0.775	(39.0 to 42.8)	0.662	(41.0 to 46.8)	0.785	(43.0 to 49.8)	0.891
Non-responders	45.5		42.0		44.5		46.0	
	(41.3 to 50.0)		(37.5 to 47.8)		(40.3 to 48.8)		(43.0 to 49.8)	
PaO_2/FiO_2 (ratio)								
Responders	314.5		337.0		323.0		330.5	
	(277.0 to 380.0)	0.683	(306.5 to 389.8)	0.817	(303.0 to 404.0)	0.341	(302.5 to 354.8)	0.967
Non-responders	333.2		310.0		310.0		316.0	
	(259.6 to 415.0)		(249.5 to 437.0)		(266.1 to 411.8)		(301.3 to 420.5)	

Abbreviations: VtC= Tidal Volume Challenge, VE= Volume Expansion, PIP= Peak Inspiratory Pressure, C_{dyn}= Dynamic Compliance, Pplat= end-Inspiratory Pressure, DP= Driving Pressure, Vt=Tidal Volume, $PaCO_2$ = Partial ressure of Carbon Dioxide, PaO_2 = Partial Pressure of Oxygen; FiO_2 = Inspiratory Oxygen

Table S2: Comparison of Ventilatory parameters After the Application of VtC and VE in Responders and Non-Responders

	before VtC (T1)	after VtC (T2)	P value	before VE (T3)	after VE (T4)	P value
PIP (cmH_2O)						
Responders	17.5 (16.3 to 19.8)	20.5 (18.3 to 23.0)	0.063	18.0 (16.0 to 20.8)	18.0 (16.3 to 20.0)	0.836
Non-responders	16.5 (15.0 to 18.8)	20.0 (17.3 to 21.8)	0.003	17.0 (16.0 to 18.0)	18.0 (15.3 to 19.0)	0.381
C_{dyn} (mL/cmH_2O)						
Responders	51.5 (34.8 to 62.2)	58.0 (42.5 to 66.0)	0.289	53.5 (37.8 to 65.9)	53.1 (46.1 to 60.8)	0.874
Non-responders	51.0 (46.0 to 56.8)	55.0 (49.0 to 63.3)	0.255	52.0 (42.5 to 58.0)	47.0 (39.3 to 56.8)	0.323
Pplat (cmH_2O)						
Responders	13.5	15.0	0.14	14.0	14.5	0.835

	(12.3 to 15.8)	(14.0 to 16.0)		(12.3 to 16.8)	(13.0 to 15.8)	
Non-responders	13.0	14.5	0.066	13.0	13.5	0.73
	(12.0 to 14.8)	(13.3 to 16.8)		(12.3 to 15.0)	(12.3 to 15.0)	
DP (cmH_2O)						
Responders	8.5	10.0	0.141	9.0	9.5	0.835
	(7.3 to 10.8)	(9.0 to 11.0)		(7.3 to 11.8)	(8.0 to 10.8)	
Non-responders	8.0	9.5	0.066	8.0	8.5	0.73
	(7.0 to 9.8)	(8.3 to 11.8)		(7.3 to 10.0)	(7.3 to 10.0)	
Vt (mL)						
Responders	382.5	513.0	0.00004	382.5	382.5	1.0
	(325.0 to 410.0	(427.5 to 540.0)		(325.0 to 410.0)	(325.0 to 410.0)	
Non-responders	375.0	493.0	0.00001	375.0	375.0	1.0
	(340.0 to 411.5)	(451.3 to 547.5)		(340.0 to 411.5)	(340.0 to 411.5)	
RR (breaths/min)						
Responders	20.0	20.0	1.0	20.0	19.5	0.795
	(16.5 to 20.0)	(16.5 to 20.0)		(16.5 to 20.0)	(16.5 to 20.0)	
Non-responders	18.5	18.0	0.864	19.0	18.0	0.894

	(16.3 to 20.0)	(16.3 to 20.0)		(18.0 to 20.0)	(16.5 to 20.0)	
$PaCO_2$ (mmHg)						
Responders	45.0	41.5	0.012	44.5	45.0	0.253
	(44.0 to 47.0)	(39.0 to 42.8)		(41.0 to 46.8)	(43.0 to 49.8)	
Non-responders	45.5	42.0	0.059	44.5	46.0	0.24
	(41.3 to 50.0)	(37.5 to 47.8)		(40.3 to 48.8)	(43.0 to 49.8)	
PaO_2/FiO_2 (ratio)						
Responders	314.5	337.0	0.334	323.0	330.5	0.924
	(277.0 to 380.0)	(306.5 to 389.8)		(303.0 to 404.0)	(302.5 to 354.8)	
Non-responders	333.2	310.0	0.733	310.0	316.0	0.438
	(259.6 to 415.0)	(249.5 to 437.0)		(266.1 to 411.8)	(301.3 to 420.5)	

Abbreviations: VtC= Tidal Volume Challenge, VE= Volume Expansion, PIP= Peak Inspiratory Pressure, C_{dyn}= Dynamic Compliance, Pplat= end-Inspiratory Pressure, DP= Driving Pressure, Vt=Tidal Volume, $PaCO_2$ = Partial Pressure of Carbon Dioxide, PaO_2 = Partial Pressure of Oxygen; FiO_2 = Inspiratory Oxygen
